# Advances in 3D peptide hydrogel models in cancer research

**DOI:** 10.1038/s41538-021-00096-1

**Published:** 2021-06-01

**Authors:** Jingwen Xu, Guangyan Qi, Weiqun Wang, Xiuzhi Susan Sun

**Affiliations:** 1 College of Food Science and Technology, Shanghai Ocean University, Shanghai, China; 2 Department of Food, Nutrition, Dietetics and Health, Kansas State University, Manhattan, KS USA; 3 Department of Grain Science and Industry, Kansas State University, Manhattan, KS USA; 4 Department of Biological and Agricultural Engineering, Kansas State University, Manhattan, KS USA

**Keywords:** Assay systems, Toxicology, Biomaterials

## Abstract

In vitro cell culture models on monolayer surfaces (2D) have been widely adapted for identification of chemopreventive food compounds and food safety evaluation. However, the low correlation between 2D models and in vivo animal models has always been a concern; this gap is mainly caused by the lack of a three-dimensional (3D) extracellular microenvironment. In 2D models, cell behaviors and functionalities are altered, resulting in varied responses to external conditions (i.e., antioxidants) and hence leading to low predictability. Peptide hydrogel 3D scaffolding technologies, such as PGmatrix for cell culture, have been recently reported to grow organoid-like spheroids physiologically mimicking the 3D microenvironment that can be used as an in vitro 3D model for investigating cell activities, which is anticipated to improve the prediction rate. Thus, this review focuses on advances in 3D peptide hydrogels aiming to introduce 3D cell culture tools as in vitro 3D models for cancer-related research regarding food safety and nutraceuticals.

## Introduction

Nutrition and safety play key roles in food improvement and innovation. Bioactive dietary compounds, such as phytochemicals, phenolic compounds, and antioxidants, have been reported to be associated with reduced risks of many chronic diseases, such as cancer (i.e., colorectal cancer), cardiovascular disease, and diabetes. For example, antioxidants can prevent carcinogen formation by scavenging free radicals in the human body to prevent oxidation-induced changes, such as lipid peroxidation, lipid radical production, and metal ion binding. Numerous antioxidants are naturally present in various vegetables, fruits, and cereal grains or are produced via bioprocessing (i.e., fermentation) or synthesis, all of which are major sources of food and nutraceutical products and applications. On the other hand, toxicity is a major concern in food development and can easily result from food additives, food contact plastics, chemicals derived from food processing, and mycotoxin-related microorganisms. Therefore, it is essential for food scientists in both academia and industry to have effective prescreening tools to predict the chemoprotective efficacy and cytotoxicity of these appropriate bioactive compounds or chemicals for use in food ingredients in a timely manner.

Traditional in vitro cell-based assays in 2D culture have been adapted for determining the efficacy and toxicity of foods with regard to anticancer activities; most studies involving prescreening are performed to understand cancer properties such as cell growth, cytotoxicity and function, and anticancer drug efficacy^1–5^. 2D culture has many well-known advantages, such as low cost, vetted protocols, and ease of cell processing, observation, and analysis. To date, more than 70% of cancer research has been conducted in 2D culture systems prior to in vivo animal study and human clinical trials^6^.

When compared with an in vivo animal model that is usually established to further determine the bioavailability, toxicity, safety, metabolic characteristics, and efficacy of drugs (i.e., bioactive compounds), however, the correlation between in vitro 2D monolayer cell culture assays and in vivo animal models has been questionable. In addition to the lack of in vivo digestive and metabolic activities in the gastrointestinal (GI) tract, stark differences between the flat surface of 2D monolayer culture systems and the 3D microenvironment in vivo are responsible for this low correlation. In the body, cells reside in an extracellular matrix (ECM), which is a 3D framework rather than a flat surface, as is a representative 2D culture system^7^. The ECM constitutes the microenvironment for cells adhesion, contains a complex mixture of proteins, sugars, growth factors, and other components surrounding the cell membrane, and acts as a scaffold that influences not only cell behavior and phenotypes but also functional properties at the protein and DNA levels^8,9^. These variations can be evidenced by various biomarkers that regulate cell responses to drugs^10–13^. For example, HeLa cells presented multiple morphologies, including rounded, mass, grape-like, and stellate shapes, in 3D PGmatrix peptide hydrogels and secreted extracellular vesicles (EVs) similar to those secreted in vivo, while these cells exhibited a flat, spindle-shaped morphology in 2D models and produced vesicles completely unlike those produced in vivo^14,15^. Although 2D models have some advantages—including simple protocols, low cost, easy access to nutrients, oxygen, chemical cues, and drugs (i.e., bioactive food compounds)—compared with 3D models or in vivo models^15^, the sensitivity of 2D models to efficacy or cytotoxicity may result from the physiological vulnerability or altered metabolic activities of cells, all of which might distort the interpretation of the results and mislead clinical application. Thus, the 2D culture method seems to not accurately predict the in vivo context^16^. Therefore, as an in vivo-like approach, advanced 3D peptide hydrogel culture systems can be more beneficial for investigating and identifying functional food ingredients with high potential for cancer inhibition or prevention, as well as for toxicity prescreening in the long term. The purpose of this review is to introduce the advances in 3D cell culture models developed in pharmaceutical discoveries in recent decades, which are anticipated to be useful references as early prediction tools in the study of food nutrients and safety in cancer research.

A variety of 3D cell culture systems, including natural polymers, biological polymers, synthetic polymers, and hybrid polymers, have been developed to provide a 3D microenvironment mimicking the natural ECM for cell growth. Among these hydrogels, peptide hydrogels, particularly self-assembling peptide hydrogels, have shown great potential to physiologically mimic the native ECM^14,17^. Cancer cells grown in the 3D culture model formed spheroids or geometric clusters that were much more similar to the tumor geometry in vivo^7,14,18–20^. The efficacy of anticancer drugs in 3D hydrogel culture has been extensively investigated in ovarian cancer^21^, breast cancer^18^, and HeLa cells^14,22^, and these studies revealed that cancer cells are more resistant to drugs in 3D culture than in 2D culture; in particular, cells maintained in 3D culture presented more interesting phenotypic and genomic manifestations than those maintained in 2D culture.

To our knowledge, little information using 3D models mimicking in vivo environment in the field of food nutrients and food safety with regard to anticancer efficacy or cancer cell phenotype at the molecular level has been available to date. Hence, this review focuses on current applications of 3D peptide hydrogel culture tools in comparison to 2D culture systems to reveal insights into the cancer cell phenotypes and efficacy of anticancer drugs in 3D models. This review also presents a small number of case studies of food bioactive compounds that inhibit cancer (i.e., liver and colon cancer) cell growth in 3D culture models. In addition, both the current limitations of 3D models as they pertain to cancer research and future research topics in food nutrition and safety areas are further discussed.

## Versatile matrices of the 3D peptide hydrogel culture model

Biomaterials used for 3D models are classified into three main categories: (1) natural or biological polymers (e.g., agarose, chitosan, chitin, collagen, fibronectin, gelatin, laminin, and hyaluronic acids), (2) synthetic polymers (e.g., polylactic acid, polyglycolide, polyethylene glycol (PEG), and polyethylene oxide)^14,18^, (3) hybrid matrices (combinations of natural and synthetic materials, e.g., PEG-conjugated proteins)^23^, (4) ECM-based matrices (e.g., mouse tumor-derived matrices such as Matrigel)^24^, and (5) peptide hydrogels (e.g., h9e, PGmatrix, RADA peptide, and PureMatrix)^14,25^. The scale of 3D culture models can be divided into three classifications: (1) macroscale (10^−^^1^–10^−^^3^ m), which is applied in tissue engineering studies, (2) microscale (10^−3^–10^−6^ m), which comprises tissues with a microscopic biomimetic tissue structure, and (3) nanoscale (10^−6^–10^−9^ m), which is used to evaluate the interaction between cells and the ECM and responses to the microenvironment by monitoring cellular activities^7^.

The use of Matrigel has been recently demonstrated for building 3D models for evaluating bioactive food compounds^24^. Matrigel is derived from mouse tumor tissue, and it has to be used on ice; thus, not only does it contain mouse tumor xenocomponents, it is also difficult to use in laboratory workflows as well as high-throughput testing^24,26^. To avoid using Matrigel, suspension or ultra-low-attachment U-bottom 96-well-plate culture methods have been used to generate 3D cell spheroids to observe the inhibitory effects of bioactive food compounds (i.e., polymethoxylated flavones, probiotics, and fucosterol) on colon cancer cells^27–29^. However, the suspension method is designed for culture of nonadherent cells such as T cells and therefore causes aggregation of adherent cells such as colon adenocarcinoma HT29 cells^27^, while 3D spheroids formed by using U-bottom 96-well plate or similar culture methods are generated by a mechanical force resulting from the physical geometry^28^. None of these methods provide a xeno-free physiologically formed 3D spheroid model. Therefore, in our review, we focus on self-assembling peptide hydrogels as scaffolds for adherent tumor cells, because peptide hydrogels are well-defined with tunable mechanical properties^18^ and high cytobiocompatibility^14^.

The main building blocks of peptide hydrogels are short peptides that self-assemble into a network of amphiphilic polymers, which are composed of over 95% volume as water, can exhibit semisolid gel features, and facilitate the transport of nutrients, oxygen, and metabolites^30^. The main advantage of peptide hydrogels is their self-assembly to form supramolecular nanostructures that mimic the natural ECM, thus supporting cell behaviors such as survival, proliferation, growth, adhesion, and invasion in a 3D space^17,18^. To date, several peptide hydrogels have been utilized in cancer research, including EAK16, RADA16 (commercial name PuraMatrix, BD Bioscience), Fmoc-FF, Fmoc-RGD, and h9e (commercial name PGmatrix, PepGel LLC). The structure, properties, advantages, and limitations of the above-mentioned peptide hydrogels are shown in Table 1.

**Table 1:** Advantages and disadvantages of available peptide hydrogels.

Hydrogel	Properties	Advantages and disadvantages	References
EAK16	Discovered in yeast; structure: 16-residue peptide with hydrophobic alanine (A) and hydrophilic glutamic acid (E) and lysine (K) residues forming a β-sheet. Types: EAK16-IV, EAK16-II, D-EAK16, L-EAK16 (made of D- or L-amino acids).	Advantages: thermally stable from 20 to 80 °C; rapidly assembles into 3D hydrogel scaffolds in physiological medium or salt solution. Disadvantage: low mechanical strength and, especially, stiffness.	Hong et al.^32^ Luo et al.^33^
RADA16	Structure: 16-residue peptide with a repeated arginine-alanine-aspartate-alanine sequence forming a β-sheet. Types: RADA16-I and RADA16II.	Advantages: forms a gel in neutral pH or physiological saline solution; facilitates cell proliferation and differentiation. Disadvantage: low mechanical strength and, especially, stiffness.	Zhang et al.^31^ Caliari and Burdick^72^ Wang et al.^73^
Fmoc-FF	Consists of a diphenylalanine (FF) peptide modified with a fluorenyl-methoxycarbonyl (Fmoc) side group representing a 3D network.	Advantages: forms a stable gel at physiological pH; has a structure and viscoelasticity similar to those of ECM, with excellent biocompatibility Disadvantages: poor mechanical properties; cytotoxic effect upon dissolution.	Ryan et al.^36^ Truong et al.^37^ Worthington et al.^38^
H9e	Combination of a hydrophobic h9 sequence and hydrophilic eD2 sequence; amphiphilic nature; can self-assemble peptides into α-helix and β-sheet structures; short recovery time; shear-thinning property.	Advantages: proper mechanical strength; remains stable as a semisolid gel at neutral pH and across a wide temperature range from 2 to 80 °C; has high similarity to the natural ECM; has no effect on cell viability.	Huang et al.^39^ Huang et al.^40^ Huang et al.^18^

The peptide EAK, which was identified in a yeast protein, has been reported to sequester more than 99% of the water content and form scaffolds in physiological media or salt solutions^31^. There are four known types of EAK16: EAK16-I^32^, EAK16-II (AEAEAKAKAEAEAKAK)^31^, D-EAK16, and L-EAK16 (composed of D- or L-amino acids)^33^. The pH affects the structure of EAK16 hydrogels; for example, at neutral pH, EAK16-IV forms a globular structure, while EAK16-II forms a fibrillar structure^32^. L-EAK16 is thermally stable from 20 to 80 °C but less stable than the D-form when exposed to enzymes such as proteases^33^. However, it has been reported that D-amino acids are sometimes toxic to cells; therefore, it is not clear whether the self-assembling D-form peptide is toxic to cells^34,35^. RADA16 is a hexadecapeptide based on the EAK16 peptide. Both EAK16 and RADA16 (commercially available as PuraMatrix) can self-assemble to provide a 3D microenvironment for studying tumor mechanisms in vitro. However, RADA hydrogel formation requires the addition of sucrose to the peptide solution and culture medium to prevent cell damage due to the acidic nature of RADA peptides.

Fmoc-FF is a system consisting of a diphenylalanine (FF) dipeptide modified with a fluorenyl-methoxycarbonyl (Fmoc) side group and is distinct from EAK16 and RADA16 due to its ability to form a stable gel at physiological pH^36^. Fmoc-FF hydrogel formation is complicated since it requires dissolving the peptide in DMSO, diluting the stock in a solution at pH 10 (Fmoc-FF) or pH 3 (Fmoc-RGD), and equilibrating the samples overnight at 37 °C to adjust for and stabilize the pH to a neutral state^37,38^. Since the stiffness of Fmoc-FF hydrogels is sensitive to pH, cell behaviors may be altered because the stiffness of Fmoc hydrogels is not mechanically similar to that of the natural ECM. In addition to these pH issues, Fmoc groups are not present in the natural ECM, which may also affect cell behaviors. Truong et al. demonstrated that the viability of the human cervical cancer cell line HeLa was affected as a function of time of Fmoc-FF gel dissolution, degradation, and leaching into the medium. However, the viability of the human colorectal cancer cell line Caco2 and human gingival fibroblasts (HGF-1 cells) was not affected by the time of Fmoc-FF gel dissolution, degradation, and leaching into the medium^37^.

**Fig. 1:**
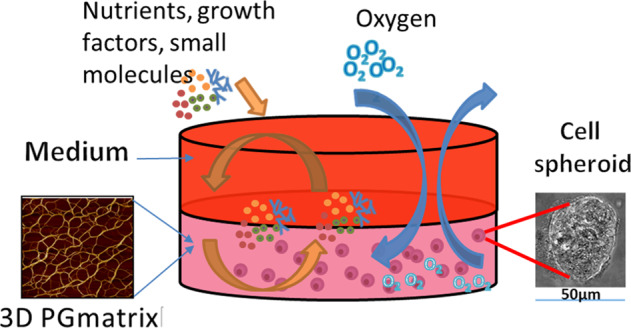
PGmatrix 3D scaffolding provides in vivo like microenvironment for cells, allows free-diffusion of nutrients, growth factors, small molecules, drugs and antioxidants (i.e, anthocyanins), and serves as an oxygen buffer. Sample image of cell spheroids physiologically formed in 3D PGmatrix culture.

The h9e peptide hydrogel is prepared by combining two native sequences, namely, those of the elastic segment of spider silk and a transmembrane segment of human muscle^39^. The h9e peptide hydrogel can remain stable as a solution or semisolid gel at neutral pH over a wide temperature range (2–80 °C)^39,40^. H9e gelation can occur at room temperature and at physiological temperatures (37 °C) due to the self-assembly of peptides without pH adjustment. Cancer cells or spheroids can be recovered (harvested) from h9e hydrogel culture for further analysis^18^. In addition, PGmatrix, a commercial product mainly containing h9e peptides, produces a high content of spheroids with high viability mimicking that observed under in vivo conditions, similar to previous reports of exosome information^14^. An example illustration of cell spheroids formed in 3D PGmatrix (mainly derived from the h9e peptide hydrogel) is shown in Fig. 1.

## Current applications of 3D peptide hydrogels for cell culture in cancer research

As mentioned above, 2D models have low predictive value in vivo; therefore, to date, the main applications of 3D peptide hydrogel culture have been to focus on cancer cell behaviors, including survival, growth, invasion, tumorigenesis, metastasis, and others, in three dimensions, and to confirm whether these cell behaviors are relevant in vivo. Cell physiological studies, cancer cell assessments, signaling pathway studies, and cell secretion studies have been reported. In addition to investigating the phenotypes of cancer cells, another striking application of 3D models is in investigating the efficacy and toxicity of bioactive food compounds for cancer prevention and inhibition for longer time periods on the month scale. Food is often consumed as a complex with multiple components processed under the action of multiple digestive enzymes as it passes from the mouth to the stomach and then into the GI tract, which consists of the duodenum, jejunum, ileum, cecum, and colon; this process presents a great challenge for in vitro studies^41^. However, most phytochemicals, phenolic compounds, and antioxidants (i.e., anthocyanins and flavonoids) are mainly detoxificated and absorbed by mucosal epithelial cells in the ileum, cecum, and colon in the GI tract^42–44^ and later transferred to the liver, for further detoxification modification as glucuronide, sulfate, and other conjugating forms^45,46^. Therefore, cells—whether cancer cells, normal cells, or inflammation-induced cells from the GI system or liver—cultured in a 3D system are a meaningful in vitro tool for preevaluation of bioactive food compounds. The 3D model illustrated in Fig. 1 can also be constructed with multiple chambers^47,48^ or easily allows coculture of multiple types of cells from different organs or culture in combination with stem cells or immune cells (i.e., macrophages). Furthermore, various detoxification enzymes can be easily added alone or in combination with food complexes into the 3D model system at designed time points to observe the efficacy and kinetics of any bioactive food compound. Since efficacy studies on bioactive food compounds in 3D peptide hydrogel models are limited, this section mainly focuses on the use of 3D peptide hydrogels for anticancer drug testing, mainly evaluating drug toxicity in comparison to that in 2D cell culture. The phenotypes and performance of cancer cells in 3D peptide hydrogel models should be important references for food scientists to select and design an effective 3D model for food toxicity and bioactive food compound studies.

### Phenotypes of cancer cells in 3D peptide hydrogel culture models

Multiple types of peptide hydrogels, such as EAK16, RADA16, Fmoc-FF, h9e, PuraMatrix, and PGmatrix, have been used to study the phenotypes of cancer cells. It has been reported that the properties of multiple types of cancer cells cultured in peptide hydrogels are superior with regard to cell morphology, survival, growth, adhesion properties, invasion potential, migration, etc. to those of cells grown in 2D models; the cell lines reported include breast cancer cells^18,25^, liver cancer cells^49,50^, ovarian cancer cells^21,51^, cervical cancer cells^14,22^, and lung cancer cells^19^. The phenotypic characteristics of cancer cells and identified biomarkers in 3D peptide hydrogels are shown in Table 2.

**Table 2:** Phenotypic characteristics of cancer cells and tested biomarkers in 3D peptide hydrogel models.

Cancer cell type	3D peptide hydrogel type	Cell behaviors in various medium and drug treatment conditions	Biomarkers	References
MDA-MB-231 breast cancer cells	RADA16, collagen I, and Matrigel	Cells are elongated in collagen I and RADA16 hydrogels but spheroid in Matrigel. Cells in RADA16 hydrogels show reduced migratory ability and tumorgenicity vs. those in collagen I hydrogels and Matrigel.	N/A	Mi et al.^25^
SMMC7221 hepatocellular carcinoma cells	RADA16, collagen I, and Matrigel	Cells form spheroids in RADA16 hydrogels and Matrigel. Cell proliferation is reduced in RADA16 hydrogels vs. collagen I hydrogels and Matrigel.	N/A	Song et al.^50^
A2780, A2780/DDP, and SK-OV-3 ovarian cancer cells	RADA16 and collagen I	Cells exhibit similar adhesion properties and invasion potential in RADA16 and collagen I hydrogels.	N/A	Yang and Zhao^21^
HO-8910PM ovarian cancer cells	RADA16, Matrigel and collagen I	Cells exhibit similar adhesion properties in RADA16 hydrogels, Matrigel and collagen I hydrogels.	Proteins related to adhesion properties: integrin β1, E-cadherin and N-cadherin.	Song et al.^51^
A549 lung adenocarcinoma cells	PuraMatrix and Matrigel	Cells form spheroids and show similar invasion potential in PuraMatrix and Matrigel.	Biomarker of invasion: cortactin	Prina-Mello et al.^19^
MCF-7 breast cancer cells	H9e	Cisplatin reduces cell viability in the hydrogel.	Biomarker of cytoskeletal function: actin; biomarker of proliferation: Ki67; biomarker of apoptosis inhibition: survivin; biomarker of active apoptosis: cleaved caspase-3.	Huang et al.^18^
HNS, UM-SSC-1, and OSC-19 head and neck squamous cell carcinoma cells	H9e	Cells form tumor-like clusters in the hydrogel; ficlatuzumab inhibits cancer cell growth.	Biomarker of epithelial-to-mesenchymal transition: vimentin	Kumar et al.^53^
ATCC CCL-2 HeLa cancer cells	PGmatrix DMEM PGD-006 PepGel LLC	Hydrogels stimulate secretion of in vivo-like extracellular vesicles (i.e., exosomes); spheroids show an in vivo tumor-like morphology.	RNA sequencing	Thippabhotla et al.^14^

The breast cancer cell line MDA-MB-231 exhibits an elongated morphology in collagen I and RADA16 hydrogels, but maintains a spheroid form in Matrigel^25^. MDA-MB-231 cells proliferate strongly in Matrigel because they are derived from mouse tumors, and Matrigel is composed of collagen, laminin, growth factors, and other molecules that promote proliferation^52^. These cells exhibit growth arrest behavior in the RADA16 hydrogel, probably because of the absence of biological “contaminants” that are present in Matrigel^25^. The migratory ability of breast cancer cells was reduced in the RADA16 hydrogel compared to the collagen I hydrogel and Matrigel^25^. The tumorigenic ability of MDA-MB-231 cells was also enhanced in collagen I and Matrigel, while cells grown in the RADA16 hydrogel formed tumors with lower weights^25^. Wu et al. isolated the human hepatocarcinoma cell line HepG2 from 2D cell culture and resuspended the cells in 20% sucrose prior to mixing with RADA16 peptide solution (RADA16-I at a peptide concentration of <1% (w/v)); the sucrose functioned to protect the cells from the acidic nature of RADA16. HepG2 cells formed more clusters, showed a higher proliferation rate, and formed larger tumors in the RADA16 hydrogel than in 2D cell culture^49^. In addition, the adhesion properties and albumin secretion of HepG2 cells were reported to be similar in both the RADA16 hydrogel and collagen I hydrogel^49^. HepG2 cells showed similar binding affinities in both RADA16 and collagen I hydrogels, indicating that the RADA16 peptide has molecular and structural characteristics similar to those of natural collagen I hydrogels. Albumin secretion was also found to be similar in RADA16 and collagen I hydrogels^49^.

The phenotypic characteristics of human hepatocellular carcinoma SMMC7221 cells—specifically, morphology, growth, proliferation, protein expression, and tumorigenic ability—were compared among cells cultured in the RADA16 hydrogel, Matrigel, and the collagen I hydrogel^50^. SMMC7221 cells formed larger spheroids in the RADA16 hydrogel and Matrigel than in the collagen I hydrogel. The expression levels of ECM proteins, such as fibronectins and laminins, were the highest in the collagen I hydrogel, followed by the RADA16 hydrogel and Matrigel. SMMC7221 cell tumorigenesis was not affected by hydrogel type, as reflected by the levels of the biomarkers VEGFA, EGF, and FGF2^50^. Ovarian cancer cells, including A2780, A2780/DDP, and SK-OV-3 cells, were cultured in RADA16 hydrogel and exhibited adhesion properties and invasion potential similar to those of cells cultured in collagen I hydrogel^21^. Collagen is a structural protein in the ECM and can promote cell adhesion. The similarity of cell adhesion properties in collagen I and RADA16 suggested that the self-assembling peptide RADA16 has structural characteristics similar to those of collagen I. Cancer cell migration was also similar in the RADA16 and collagen I hydrogels, which further indicated that the RADA16 hydrogel has biological functionalities similar to those of collagen I^21^. In addition, the RADA16 hydrogel enhanced the invasion potential of ovarian cancer cells^21^. Similar results indicated that there were no significant differences in the adhesion properties of HO-8910PM ovarian cancer cells cultured in RADA16^51^, Matrigel, and collagen I, based on the protein expression levels of integrin β1, E-cadherin, and N-cadherin, as measured via immunohistochemical staining. Quantification of integrin β1, E-cadherin, and N-cadherin expression was further determined by Western blot analysis, and the expression of each protein varied in different 3D hydrogels^51^. The behaviors of A549 lung adenocarcinoma cells were studied in PuraMatrix (commercial name of RADA16) and Matrigel, and the cell morphology was shifted toward a 3D spheroid geometry^19^. A549 cell viability was reported to be increased in cells cultured in PuraMatrix compared to those in 2D cell culture, but was not elevated in cells cultured in Matrigel^19^. Cortactin, a biomarker of invadopodia that is responsible for A549 cell invasion, was detected through Western blotting in both hydrogels, indicating that the invasion potential of A549 cells was similar in PuraMatrix and Matrigel^19^.

The human epithelial breast cancer cell line MCF-7 was cultured in a 3D h9e peptide hydrogel to compare its morphology, survival, proliferation, and apoptosis to those of MCF-7 cells in 2D cell culture^18^. MCF-7 cells formed tumor-like spheroids in the h9e hydrogel and grew slower, possibly because of either the transition from the 2D to the 3D environment or the effects on the cell growth rate in the 3D environment, which is commonly considered to be more similar to in vivo conditions. However, cell viability in the h9e hydrogel remained similar to that in 2D cultures^18^. Spheroids generated from HeLa (CCL-2) cervical cancer cells were cultured in 3D PGmatrix (commercial name of the h9e hydrogel), and the growth rate of HeLa cells was slower than that in 2D culture^22^. In addition, the invasiveness of head and neck squamous cell carcinoma (HNSCC) cells was studied by culturing HNSCC cells in the upper chamber of a transwell insert coated with a PGmatrix and observing the number of cells that accumulated in the lower chamber below the transwell insert^53^. The researchers found that HNSCC cells migrated and invaded through the PGmatrix and that these migration and invasion activities were inhibited by the hepatocyte growth factor (HGF) inhibitor ficlatuzumab^53^.

The literature has multiple reports on the phenotypic characteristics of cancer cells in versatile 3D peptide hydrogels with regard to morphology, survival, proliferation, growth, adhesion, invasion, migration, and other characteristics. It has been suggested that more cancer cell types in addition to the above-mentioned cancer cell types be evaluated in 3D peptide hydrogels. In 3D peptide hydrogels, cells form tumor-like spheroids or clusters that are similar to those in vivo. The progression of cancer cells in 3D peptide hydrogels is cell- and hydrogel-dependent with regard to matrix stiffness and ECM components such as growth factors. However, little is known about biomarkers of cell adhesion, invasion, migration, and apoptosis. Thus, more studies should focus on identifying biomarkers of cell behaviors. In addition, the phenotypes of cancer cells can be further investigated in vivo to study the correlation between in vitro 3D peptide hydrogel culture systems and in vivo models.

### Cell secretion and DNA transfection studies in 3D peptide hydrogel culture models

Cell secretions, such as EVs, were recently studied in 3D peptide hydrogels. For example, HeLa (CCL-2) cells cultured in 3D PGmatrix from PepGel LLC (mainly derived from h9e hybrid peptides) showed multiple in vivo-like characteristics, such as spheroids with rounded, mass, grape-like, and stellate morphologies, which have not been observed in 2D culture^14^. EVs (e.g., exosomes) secreted by cells grown in 3D PGmatrix and 2D culture were studied by RNA sequencing analysis with regard to secretion dynamics and essential signaling molecular content (RNA and DNA). Exosomes isolated from a HeLa spheroid culture in 3D PGmatrix were smaller than those isolated from 2D culture, and cells cultured in the 3D system showed higher exosome production activity. The RNA profile of the exosomes isolated from cells cultured in 3D PGmatrix showed 96% similarity to that of in vivo exosomes isolated from the circulating plasma of a cervical cancer patient^14^. The RNA profile of the exosomes isolated from the 2D cell culture was not similar to that of in vivo exosomes^14^. It was also demonstrated by DNA sequencing analysis that compared to the in vivo conditions, the 3D PGmatrix did not affect the genomic information relayed by secreted EVs in terms of culture and growth conditions, showing the potential of 3D PGmatrix for replacing in vivo studies of cancer phenotyping and drug testing^14^. PGmatrix was also reported to be applied via 3D printing to a lab-on-chip model, which allows DNA electroporation^48^. One group demonstrated that DNA was successfully transiently transfected into HeLa (CCL-2) cells through the cell membrane without affecting cell viability and transfection efficiency. Therefore, this advanced microassembly model shows the potential of the PGmatrix for use in microfluidic electroporation systems for tissue engineering^48^.

### Drug efficacy testing in 3D peptide hydrogel culture models

To date, a variety of cancer cells cultured under 3D conditions have shown higher drug resistance than those cultured in 2D conditions, including breast cancer cells^18,54^, ovarian cancer cells^21,51^, cervical cancer cells^22,55^, and other cells. The results of drug efficacy assays are significantly affected by the hydrogel composition. The anticancer drug ellipticine was tested in the non-small-cell lung cancer cell line A549 and the breast cancer cell line MCF-7 cultured in various EAK16 peptide hydrogels, including EAK16-II, EAK16-IV, and EFK16-II^54^. Ellipticine inhibited cancer cell growth in the EAK16-II and EAK16-IV hydrogels, resulting in decreased cell viability; however, EAK16-II and EAK16-IV cultures treated with a water-based ellipticine solution were not stable, leading to less-accurate toxicity testing^54^. The complexes comprising the EFK16-II hydrogel with ellipticine were stable, while the original toxicity in these complexes was lower than that in complexes containing EAK16-II and EAK16-IV. Increased hydrophobicity of the peptides significantly increased the stability of the complex. In this study, gels were formed by mixing the peptide solution and culture medium. Cell viability was determined by an MTT assay; however, the gelation time prior to testing was long (i.e., overnight)^54^.

It has also been reported that hydrogel matrix stiffness influences cellular properties such as morphology, metabolism, and function, as well as the response to anticancer drugs^56^. It was demonstrated that the anticancer drug paclitaxel inhibited the growth of MDA-MB-435S breast cancer cells cultured in the RADA16 hydrogel and that the efficacy increased as the peptide concentration increased^57^. Studies also showed that increased 3D RGD matrix stiffness and cell–matrix adhesion sites resulted in increased resistance of human U-87 and U-251 glioblastoma cells to acrylamide and cadmium chloride^58^. In addition, the increases in 3D RGD stiffness and adhesion sites increased the resistance of human hepatocellular carcinoma cells to the anticancer drugs paclitaxel, cisplatin, and 5-FU^59^.

In a 3D model with the h9e peptide hydrogel, the inhibitory effect of cisplatin on breast cancer MCF-7 cells was detected via immunofluorescence staining of a small number of biomarkers, such as actin, Ki67, survivin, and cleaved caspase-3; the levels of these biomarkers corresponded to the effects of cisplatin on cytoskeletal dynamics, cell proliferation, and apoptosis inhibition and activation^18^. It was also demonstrated that actin in MCF-7 cells was more filamentous in 2D culture but more globular in 3D h9e hydrogel culture. Ki67 was only detected in MCF-7 cells with unchanged nuclei in the 3D h9e hydrogel. Survivin was detected around a few intact nuclei; however, cleaved caspase-3 was widely detected in cisplatin-treated cells in the 3D h9e hydrogel model^18^. The inhibitory effects of ficlatuzumab on HNSCC cell lines, including HNS, UM-SSC-1, and OSC-19, via c-Met receptor-mediated proliferation, migration, and invasion, were observed in the PGmatrix hydrogel (derived from h9e)^53^. Ficlatuzumab inhibited HNSCC cell growth mediated by tumor-associated fibroblast (TAF)-secreted HGF by blocking vimentin expression induced by conditioned medium derived from TAFs. Vimentin is a biomarker for epithelial-to-mesenchymal transition in cancer cells and downregulation of the mitogen-activated protein kinase signaling pathway^53^. In another study, 3D PGmatrix was reported to facilitate the diffusion of camptothecin throughout the gel, which resulted in effective dose-dependent inhibition of HeLa cell proliferation and viability^22^. PGmatrix was also used for controlled release of the BSA-cisplatin complex into HeLa cells^55^. The efficacies of drugs on cancer cells cultured in 3D peptide hydrogel models are shown in Table 3.

**Table 3:** Efficacy of drugs or antioxidants in 3D peptide hydrogels vs. 2D culture.

Cancer cell type	3D peptide hydrogel	Drug/antioxidant	Response to drugs	References
A2780, A2780/DDP, and SK-OV-3 ovarian cancer cells	RADA16	5-FU, paclitaxel and curcumin	Cells were more resistant to drugs in RADA16 vs. 2D culture.	Yang and Zhao^21^
HO-8910PM ovarian cancer cells	RADA16	Cisplatin and paclitaxel	Cells were more resistant to drugs in RADA16 vs. 2D culture.	Song et al.^51^
MCF-7 breast cancer cells	H9e	Cisplatin	Cisplatin inhibited cell growth in h9e peptide hydrogels.	Huang et al.^18^
HeLa cervical cancer cells	PGmatrix	Camptothecin	Camptothecin reduced cell proliferation and viability in h9e peptide hydrogels.	Liang et al.^22^
ONS-76 human medulloblastoma cells	3D Max8 β-hairpin	Cisplatin, vismodegib, and histone deacetylase inhibitors	Cells in 3D hydrogels were more resistant to cisplatin and vismodegib vs. those in 2D culture but were more sensitive to histone deacetylase inhibition vs. those in 2D culture.	Worthington et al.^74^
HepG2 liver cancer cells and SW480 colorectal cancer cells	H9e	Chlorogenic acid	HepG2 and SW480 cells showed greater resistance to chlorogenic acid in h9e peptide hydrogels vs. 2D culture.	Xu et al.^61^

### Discoveries of bioactive food compounds in 3D peptide hydrogel culture models

3D spheroid models have been recently used for bioactive food compound studies with comparisons to traditional 2D models. Matrigel was used to build a 3D spheroid model to study the anticancer effects of iturin A, a well-known antifungal compound produced by *Bacillus subtilis*, on HepG2 liver cancer cells^24^. The 3D spheroid model simulated a similar in vivo microenvironment, and as a result, a higher inhibitory concentration of iturin A was observed in 3D culture than in 2D culture^24^. 3D models of mechanically formed spheroids, such as nonadherent U-bottom well plates or similar methods, have also been recently used to study the anticancer effects of bioactive food compounds, for example, the effects of polymethoxylated flavones derived from citrus peel on colorectal cancer cells (HT29)^27^, the microalgal strain *Picochlorum* sp. RCC486 on breast cancer cells (MDA-MB-231)^60^, the probiotic bacterium *Lactobacillus fermentum* on colorectal cancer cells (HCT116)^28^, and the seaweed compound fucosterol on colorectal cancer cells (HT29 and HCT116)^29^. In all these studies, 3D spheroids showed higher resistance to the anticancer effects of bioactive compounds and greater proliferation inhibition than cells in 2D models.

**Fig. 2:**
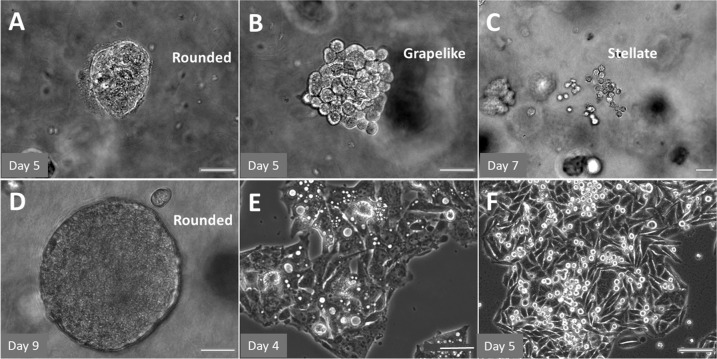
Physiological spheroids formed in 3D PGmatrix culture. Spheroids of SW480 colorectal cancer cells (**A** rounded, **B** grape-like, **C** stellate) and spheroids of HepG2 liver cancer cells (**D** rounded) cultured in 3D PGmatrix and 2D cultures of HepG2 (**E**) and SW480 cells (**F**), scale bar, 20 µm.

To the best of our knowledge, little is known regarding the anticancer efficacy studies of using 3D peptide hydrogel model systems in evaluating food nutrients and safety. Chlorogenic acid (CGA) has been shown to have inhibitory effects on liver and colon cancer tumor growth in 2D model studies; therefore, it is a commonly used antioxidant in food- and nutraceutical-related products. Recently, the liver cancer cell line HepG2 and colorectal cancer cell line SW480 were studied in a 3D peptide hydrogel (PGmatrix, PepGel LLC) model; these two cell lines formed spheroids and presented in vivo tumor-like morphologies in 3D PGmatrix hydrogels (Fig. 2A–D), while these cells were flat with a spindle-like morphology in a 2D model (Fig. 2E, F)^52^. The inhibitory effects of CGA on HepG2 and SW480 cells were investigated in a 3D h9e peptide hydrogel (i.e., PGmatrix)^61^. The 3D h9e peptide hydrogel culture system favored the growth of both cell lines, which was indicated by an extended exponential phase, and increased cell proliferation compared with that in 2D culture^61^. The inhibitory effect of CGA on HepG2 and SW480 cells was dose-dependent in both the 2D and 3D h9e hydrogel cultures; however, both HepG2 and SW480 cells were more resistant to CGA treatment in the 3D h9e hydrogel culture than in the 2D culture^61^. As a result, after CGA treatment, cells in the 3D h9e model recovered, while cells in the 2D model started dying, indicating that CGA has a diminished impact on the proliferation of HepG2 and SW480 cells in the 3D system^61^. It has been clearly demonstrated that the 3D model is superior to the 2D model in terms of mimicking the in vivo microenvironment. However, further investigations at the molecular level are warranted to correlate the effects of chemopreventive agents (i.e., CGA) on cancer cells (i.e., HepG2 and SW480) in 3D models with those in in vivo animal studies or human clinical trials.

The cytotoxic activity of antioxidants is determined by the number and position of hydroxyl and methoxy groups within the structure. For example, the cytotoxic effect of flavonoids is linked to the presence of C2–C3 double bonds, 4-carbonyl groups, and ortho-hydroxylation on the B ring^62^. The mechanisms involved in flavonoid-mediated cytotoxicity include proteasome inhibition, fatty acid synthesis inhibition, cell cycle arrest induction, and p53 accumulation. To date, little is known about the effect of antioxidant-mediated cytotoxicity on cancer cells in 3D culture models. Herein, it is highly recommended to investigate the cytotoxic effects of antioxidants on regulating cells in a 3D peptide hydrogel model and reveal the cytotoxic mechanisms and correlations in vivo.

## Summary

2D models have been extensively used in food nutrition and food safety studies. 2D model methods are simple, inexpensive, and provide easy access to nutrients, oxygen, chemical cues, and bioactive food compounds^15^. Thus, cells cultured in 2D models are more sensitive to drugs than those cultured in 3D models. For a given bioactive compound (i.e., anthocyanin), a 2D model may be useful as a rapid prescreening method to identify relevant effects of compound preparation methods or carriers (i.e., nanoparticles) for bioavailability studies. However, a controversial study reported that when cultured in a 3D Max8 β-hairpin peptide hydrogel, the human medulloblastoma cell line ONS-76 was more sensitive to cisplatin and vismodegib than when cultured in a 2D system, although the study also stated that ONS-76 cells in 3D hydrogel culture were more resistant to chemotherapeutic histone deacetylase inhibition^63^. This result indicated that cells grown in 3D culture could exhibit a more realistic response (either sensitivity or resistance) to a given drug than those grown in 2D culture. The toxicity and efficacy of food-related chemicals and anticancer bioactive food compounds observed in 3D peptide hydrogel models have not been fully confirmed in in vivo animal models, although a few case studies have shown that the efficacy and toxicity of drugs tested in 3D peptide hydrogel models are more similar to those reported clinically^14,63–65^. Furthermore, the results of drug testing in rodent studies were reported to be only 43% consistent with those in human clinical trials^66^. This failure mainly results from unaccepted and undesired drug toxicity due to different responses in humans and animals; thus, the cytotoxic effects on cancer cells need to be further clarified. Therefore, researchers are encouraged to more clearly understand the cell response to food compounds in 3D peptide hydrogels at the biomarker level to identify cell behaviors and establish realistic correlations of data from 3D peptide hydrogel models with in vivo or clinical data to elucidate the cytotoxic mechanism leading to enhanced tumor progression.

**Fig. 3:**
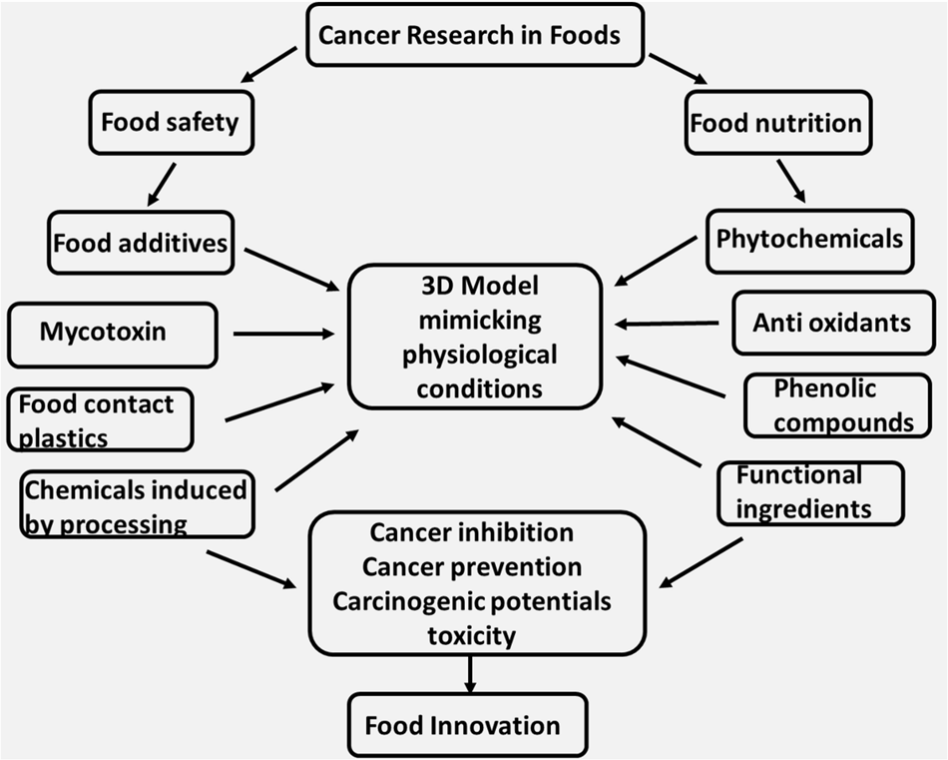
3D model mimicking in vivo microenvironment enables predictive-efficency of efficacy and toxicity analysis of bioactive food compounds and chemicals. Sample image highlighting the applications of in vitro 3D model systems in cancer research pertaining to food safety and food nutrition.

To date, little is known about cancer research in 3D peptide hydrogel models with regard to food nutrition and food safety. As shown in Fig. 3, food innovations for inhibiting and preventing cancer heavily involve aspects of food nutrition. There are many varieties of anticancer and nutritional compounds, such as phytochemicals, antioxidants, phenolic compounds, and functional ingredients. How to effectively select these compounds to achieve the targeted goals relies heavily on prescreening tools. Among the 3D peptide hydrogel models discussed above, RADA16 (PuraMatrix, formerly BD Science, currently Corning) and h9e (PGmatrix, PepGel LLC) have been used for various studies and have shown promising results. However, the major drawback of PuraMatrix is its acidic nature, which is harmful to cells during encapsulation. To protect cells from these acidic conditions, they are often preencapsulated with sucrose solution before mixing with PuraMatrix solution, and gelation is then triggered by increasing the pH to neutral (~7.0), which is achieved by adding cell medium on top of the PuraMatrix-cell solution. The h9e peptide hydrogel and PGmatrix from PepGel LLC (mainly derived from h9e) showed the most promise for mimicking in vivo conditions in an in vitro 3D model, because cells cultured in these systems exhibited in vivo-like phenotypes at the RNA level. In addition, cells and spheroids cultured in 3D PGmatrix can be easily harvested for further analysis. Although both PGmatrix and PuraMatrix are xeno-free and well-defined matrices, PGmatrix can be easily manipulated at room temperature or under physiological conditions, which makes PGmatrix a possible option for either laboratory setting or high-throughput processes.

These advanced 3D model tools can also be used for cytotoxicity-related food safety research, as shown in Fig. 3, which is another important aspect of food innovation and improvement. There are numerous chemicals and compounds derived from food additives (i.e., silicon-related), food processing (i.e., acrylamide), food contact packaging materials (i.e., plastics, bisphenol), and mycotoxins. These 3D models would be effective in rapidly discovering the toxicity level or carcinogenic potential of any existing or new compound in food applications.

## Conclusion and outlook

One challenging issue in using traditional 2D models is their low correlation with animal models, while establishing animal models is time-consuming and expensive. In addition, another great challenge is determining the long-term impact of new and existing food ingredients or bioactive compounds on carcinogen formation in animal models within the testing time frame. However, well-defined in vitro 3D models mimicking in vivo conditions may allow more accurate characterization of variations at the cellular, protein, and DNA levels induced by bioactive food compounds, ingredients, or additives. These variations may be highly predictive of the potential of carcinogen formation in the long term or of cancer development after 40–60 years. Therefore, animal models may constitute a bottleneck for examining or reexamining the safety and efficacy of vast amounts of new and existing bioactive food compounds, ingredients, and additives in a timely manner. 3D peptide hydrogels have been used to study cancer cell phenotypes and the anticancer efficacies of drugs, and these models could be adapted for bioactive food compound studies to obtain physiologically relevant efficacy and cytotoxicity data during the food discovery process. Since bioactive compounds are often consumed together with food complexes, the fate of any bioactive compound in the digestive system from the mouth to the GI tract greatly affects its ultimate bioavailability^41–46^, which should be taken into consideration for establishing 3D model protocols for food safety and nutrition research. The 3D model allows culture of cancer cells in the presence of normal cells and coculture of multiple cell types in the presence of immune cells, for example, which is necessary for correlation with in vivo situation. Examples of potential research opportunities for readers to reference include but are not limited to those suggested: (1) to establish highly predictive 3D peptide hydrogel models for assessment of food toxicity and bioactive food compounds, statistical correlation analyses should be conducted in parallel between 3D peptide hydrogel models and animal studies as well as clinical trials. Key variables highly recommended to be considered in these studies include (1) a complexity of food composition mimicking that of real food, (2) the incorporation of physiologically formed 3D spheroids, and (3) the fates of bioactive food compounds in the GI tract, digestive enzymes, metabolic factors, and immune defense factors. The main advantage of 3D peptide hydrogels is that they can be easily built into customized models so that these factors can be taken into consideration during 3D model system design, (2) a better understanding of the underlying mechanism by which bioactive food compounds (i.e., antioxidants) inhibit or prevent carcinogen formation at the cellular and DNA levels is needed. Many tumors form after a very long time frame; for example, colon carcinogenesis can be initiated after 10–30 years of local rectal inflammation. Inhibiting rectal inflammation is anticipated to prevent carcinogenesis over the long term, and this possibility can be investigated by designing 3D inflammation models or 3D target gene knockout models. (3) Another highly challenging research opportunity in food safety and nutrition is to establish the predicted equivalent intake amounts (e.g., for the effects of bioactive food compounds on cancer inhibition or food toxicity on carcinogen formation) derived from 3D models that are relevant for animal studies and for clinical trials and uses. (4) 3D spheroid models can be powerful tools to evaluate the long-term impacts of food ingredients, bioactive food compounds, and food additives on carcinogenesis in vital organs, particularly digestive organs such as the liver, colon, and pancreas. (5) The 3D spheroid model can also be a good system in which to evaluate the long-term impact of chemicals or compounds derived from food packaging and processing. For a given bioactive food compound, there are many variables affected by its preparation and processing methods that influence its bioavailability, efficacy, and toxicities; these variables include but are not limited to particle size, purity, and crystallinity, cross-linking, and entanglement. For a given compound, delivery methods (i.e., nanoparticles) to improve the bioavailability of the compound are another emerging research area^67,68^. Collectively, these factors generate a considerable bottleneck. 2D models may be useful for rapid initial screening to identify relevant effects for a given compound, and 3D peptide hydrogel models can then be useful for further studies on efficacy or cytotoxicity to obtain more predictive data before an in vivo animal model is used. These combination testing strategies also need to be investigated and standardized. (6) Finally, 3D spheroid models can more closely mimic in vivo intercellular interactions by allowing coculture of multiple types of cells. For example, chemopreventive agents can be evaluated in a 3D cancer cell and active macrophage cell coculture model, allowing a mechanistic understanding of inflammatory cytokine-induced anticancer effects in vivo and providing reliable chemotherapeutic approaches.

Applications of peptide hydrogels are currently not limited to cancer research. For example, h9e hydrogel hybrids such as PGmatrix have been used with a variety of organ cell lines to mimic organoid systems, create organ-on-chip or body-on-chip models^47,48^, select and separate stem cells^69^, hemorrhage controls^70^, and deliver drugs and biological compounds (i.e., vaccines) in vivo^39,71^. In the future, 3D peptide hydrogels can be used as in vitro models mimicking in vivo conditions for food toxicity and bioactive food compound screening to accelerate the pace of anticancer compound discovery or as carriers for in vivo delivery of biological or nutritional compounds or even cells to improve human health.

## Data Availability

Data available on request from the authors.
