# Author Correction: Advances in 3D peptide hydrogel models in cancer research

**DOI:** 10.1038/s41538-021-00110-6

**Published:** 2021-08-26

**Authors:** Jingwen Xu, Guangyan Qi, Weiqun Wang, Xiuzhi Susan Sun

**Affiliations:** 1 College of Food Science and Technology, Shanghai Ocean University, Shanghai, China; 2 Department of Food, Nutrition, Dietetics and Health, Kansas State University, Manhattan, KS USA; 3 Department of Grain Science and Industry, Kansas State University, Manhattan, KS USA; 4 Department of Biological and Agricultural Engineering, Kansas State University, Manhattan, KS USA

**Keywords:** Assay systems, Toxicology, Biomaterials

Correction to: *npj Science of Food* 10.1038/s41538-021-00096-1, published online 01 June 2021

The original version of this Article contained an error in the author affiliations.

The affiliation for Weiqun Wang incorrectly read ‘College of Food Science and Technology, Shanghai Ocean University, Shanghai, China’. The correct affiliation is ‘Department of Food, Nutrition, Dietetics and Health, Kansas State University, Manhattan, KS, USA’.

This has now been corrected in both the PDF and HTML versions of the Article.

